# Some Women Who Lost Their Heads

**Published:** 1919-11-01

**Authors:** 


*To the Editor of* The Hospital.

### "Some Women who Lost their Heads."

*To the Editor of* The Hospital.

Sir,—A good many reckless statements are being circulated by so-called nurses and others, including the "unfair competition of hospitals keeping private staffs and undercutting trained nurses," and "the flooding of the nursing market with partially trained Voluntary-Aid workers." Common Sense requires evidence of this and the names of the hospitals in question. Where, too, are such Voluntary-Aid workers to be met with? Miss Macdonald's statement, "thousands of nurses are out of employment because they cannot secure a living wage," produced no evidence for her assertion. Hers are but statements. What we demand on behalf of every independent woman worker belonging to the nursing body are facts. Let Miss Macdonald produce a list of the 1,000 or even 100 fully trained nurses' names and addresses who are in the position she asserts, or make ample apologies for wild statements and for losing her head. Common Sense.

